# Rapid detection of *Mycobacterium bovis *DNA in cattle lymph nodes with visible lesions using PCR

**DOI:** 10.1186/1746-6148-3-12

**Published:** 2007-06-13

**Authors:** G Michael Taylor, Danny R Worth, Si Palmer, Keith Jahans, R Glyn Hewinson

**Affiliations:** 1 Centre for Microbiology and Infectious Diseases, Flowers Building, Imperial College, Armstrong Road, London SW7 4AZ, UK; 2 TB Diagnostic Section, Veterinary Laboratory Agency, Woodham Lane, New Haw, Addlestone, Surrey KT15 3NB, UK

## Abstract

**Background:**

We have evaluated a sensitive screening assay for *Mycobacterium tuberculosis *(MTB) complex organisms and a specific assay for detecting *Mycobacterium bovis *DNA in lymph nodes taken from cattle with evidence of bovine tuberculosis. Underlying these series of experiments was the need for a versatile DNA extraction protocol which could handle tissue samples and with the potential for automation.

The target for the screening assay was the multi-copy insertion element IS*1081*, present in 6 copies in the MTB complex. For confirmation of *M. bovis *we used primers flanking a specific deletion in the genome of *M. bovis *known as region of difference 4 (RD4). The sensitivity and specificity of these PCRs has been tested on genomic DNA from MTB complex reference strains, mycobacteria other than tuberculosis (MOTT), spiked samples and on clinical material.

**Results:**

The minimum detection limits of the IS*1081 *method was < I genome copy and for the RD4 PCR was 5 genome copies. Both methods can be readily adapted for quantitative PCR with the use of SYBR Green intercalating dye on the RotorGene 3000 platform (Corbett Research).

Initial testing of field samples of bovine lymph nodes with visible lesions (VL, n = 109) highlighted two shortfalls of the molecular approach. Firstly, comparison of IS*1081 *PCR with the "gold standard" of culture showed a sensitivity of approximately 70%. The sensitivity of the RD4 PCR method was 50%. Secondly, the success rate of spoligotyping applied directly to clinical material was 51% compared with cultures. A series of further experiments indicated that the discrepancy between sensitivity of detection found with purified mycobacterial DNA and direct testing of field samples was due to limited mycobacterial DNA recovery from tissue homogenates rather than PCR inhibition. The resilient mycobacterial cell wall, the presence of tissue debris and the paucibacillary nature of some cattle VL tissue may all contribute to this observation. Any of these factors may restrict application of other more discriminant typing methods.

A simple means of increasing the efficiency of mycobacterial DNA recovery was assessed using a further pool of 95 cattle VL. Following modification of the extraction protocol, detection rate with the IS*1081 *and RD4 methods increased to 91% and 59% respectively.

**Conclusion:**

The IS*1081 *PCR is a realistic screening method for rapid identification of positive cases but the sensitivity of single copy methods, like RD4 and also of spoligotyping will need to be improved to make these applicable for direct testing of tissue extracts.

## Background

Bovine tuberculosis is a chronic granulomatous disease mainly affecting lymph node and lung tissues of cattle. It is caused by *Mycobacterium bovis*, a member of the *Mycobacterium tuberculosis *complex group of bacteria. At the genome level, *M. bovis *shares 99.95% identity with *Mycobacterium tuberculosis*, the agent of human tuberculosis [1].

The various ecotypes of *M. bovis *have a wide host range [2] and can infect a variety of species such as badgers [3,4], deer [5], smaller mammals [6] and diverse free-living and domesticated species [2,7]. More exotic species may also be at risk through the diversification of farming practices [8,9]. Badgers and deer can become reservoirs of disease, making eradication from the countryside difficult, although the risks posed by each species are still the subject of research and debate. Humans are rarely affected, but people in some occupations such as veterinarians, farmers and abattoir workers may be more at risk [10-12]. In the UK, human cases tend to be isolated events with no maintenance of the disease in the population [13].

Bovine tuberculosis (bTB) is not uniformly distributed throughout Great Britain but concentrated in the south-west of England and Wales. Broadly, these hot-spot areas of disease match areas of highest cattle density in UK herds. Cattle-to-cattle transmission is therefore a likely significant route of infection which may be exacerbated by the movement of infected cattle [14]. Surveillance testing of cattle for bTB is carried out using the tuberculin skin test. Positive reactors are slaughtered and examined *post mortem *when tissues are taken for confirmatory testing by culture. Herds in which a breakdown has occurred are subject to repeat testing every 60 days until two tests are clear. Approximately 6% of cattle herds were under restriction at some point in 2005 because of a bTB incident. In the same year, approximately 30,000 cattle (reactors, incomplete reactors and close contacts) were slaughtered; the financial cost of testing and in compensation payments to farmers amounted to £88 million. The trend is for confirmed new incidents to increase by 18% per year posing a growing risk to animal health and welfare and an increasing financial burden for the taxpayer [15].

Routine culture and histology of lymph nodes both with and without visible lesions (VL and NVL), are undertaken to isolate and type the causative strain of the organism. In 2005, the last complete year for which figures are available, 18,696 samples were sent for culture to the Veterinary Laboratory Agency (VLA). Of these, 5,507 were from cases with VL and 13,189 from NVL cases. A further 792 samples with VL were found during surveillance at the slaughterhouse. In these cases, culture supported by histological examination is used to confirm or rule out infection with *M. bovis*. Culture results take at least 3 weeks but usually take up to six weeks for paucibacillary specimens. The diagnosis is made on morphological grounds by characteristic appearance of the mycobacterial colonies on various growth media. DNA prepared from cultures is used for molecular typing studies including spoligotyping [16] and variable nucleotide tandem repeat (VNTR) typing [13,17].

In this setting, the development of a reliable and rapid screening test would be of great help in the control of the disease and in specific situations such as faster confirmation of bovine TB infection in slaughterhouse cases. Polymerase chain reaction (PCR) methods offer great potential in this respect and several methods, including real-time PCRs have been evaluated for bTB applications [18-21]. These have been used in research applications and epidemiological studies, but have yet to make significant impact on diagnostic procedures in the UK. With information gained from the *M. bovis *sequencing project [1,22], we have evaluated two PCR methods for possible diagnostic use; a sensitive screening method and a specific confirmatory test for bTB. Two separate studies were undertaken on cattle lymph nodes with VL and the results compared. Study 1 comprised of 109 lymph node tissue samples with VL received in 2003 by the VLA, Weybridge for *M. bovis *testing. Based on the PCR results obtained from study 1, modifications were made to the DNA extraction procedure and the effects of these were evaluated in a second study of further 95 VL samples received during 2004.

## Results

### Tissue culture

*M. bovis *positive isolates were identified according to their growth characteristics and appearances on the Lowenstein-Jensen base (LJ), LJ plus glycerol (LJG), LJ plus pyruvate (LJP) and 7H11 slopes. The organisms grow poorly or not at all on LJG. Rough, opaque colonies appear on the other media. These float off when the slope is tilted and the inoculum liquid residue at the bottom of the tube washes across the surface.

### Heat inactivation trial of *M. bovis *extracted in NucliSens™ buffer

No growth was observed in either the *M. bovis *standard AF61/2122/97 or in the cattle sample (AF61/2834/02) when these were taken into the guanidinium lysis buffer (heated or unheated). In contrast, growth on 7H11 medium was observed in the control aliquots of AF61/2122/97. Lysis buffer completely inactivated *M. bovis *cells over 12 hours at 4C without the need for heat inactivation. The conclusion is that samples from cattle VL tissues treated in this way can be safely processed outside of a Cat III area.

### PCR minimum detection limits

The IS*1081 *method was the most sensitive, detecting as little as 2.35 fg DNA (0.5 genome copies), whereas the RD4 method, which amplifies a single copy target, was 10 times less sensitive at 23.5 fg (5 genome copies).

### Validation of IS*1081 *and RD4 PCR methods

The IS*1081 *method detected all of the MTB complex strains tested including *M. canetti, M. tuberculosis *(H37Rv, H37Ra, CDC1551), all microti reference and field strains, the *M. bovis *strain AF2122/97, BCG Pasteur and the VLA panel of 10 major spoligotypes. MOTT strains were not amplified.

The RD4 method detected *M. bovis *AF2122/97, BCG Pasteur and the VLA panel of 10 different spoligotypes. No products were amplified from other MTB complex members (including the fur seal isolate) or any of the MOTT tested.

### PCR of field samples

Study 1. One hundred and nine samples were extracted using the procedures described below. Cultures were set up and compared with PCR methods for bovine DNA (*cyt b*) and the *M. bovis *PCR methods using IS*1081 *and RD4. In this study, 98/109 samples were found to be culture positive and grew *M. bovis*. Of these, 69 were positive for IS*1081*, a sensitivity of 70.4%. Three of the 11 culture negative samples were IS*1081 *PCR positive. The RD4 PCR detected 49 of the 98 culture positives (50%). The same 3 culture negative samples were also positive by RD4 PCR. The reasons for the low PCR sensitivity were investigated. A modified hemi-nested IS*1081 *PCR method was used to re-amplify DNA from all 109 samples. The sensitivity rose by only 5% to 75.4% detected, suggesting that first round PCR was efficient. The *cytb *housekeeper gene was successfully amplified from all tissues. These observations implied that the level of DNA recovery from mycobacteria was probably the cause for the low pick-up rate. A number of experiments were performed to find the cause. Spiking of control cattle tissue with different numbers of BCG cells showed that recovery from these measured by real-time PCR was as low as 22% of the theoretical added colony forming units (cfu), determined by culture.

Three modifications were tried to improve the extraction efficiency. These were 1. Bead beating (Ribolyser, Hybaid), 2. Sonication and 3. Inclusion of an additional step of 3 freeze-thaw cycles of crude extracts in liquid nitrogen. For sonication, the Elma T 460/H ultrasonic bath operating at 35 KHz was used (Elma, Singen, Germany). Sonication for 15 minutes resulted in improved recovery in some samples but resulted in PCR failures of known positives and was therefore abandoned. This may have been due to variability between tissue homogenates. Bead beating was effective but more expensive and tubes were prone to leakage, raising issues of cross-contamination for routine diagnostic use. In pilot experiments using samples spiked with known numbers of *M. bovis *cells determined by culture, freeze-thawing improved recovery of added DNA by 3 -fold and could be achieved without opening the tubes.

### Study 2 PCR versus culture

**Figure 2:**
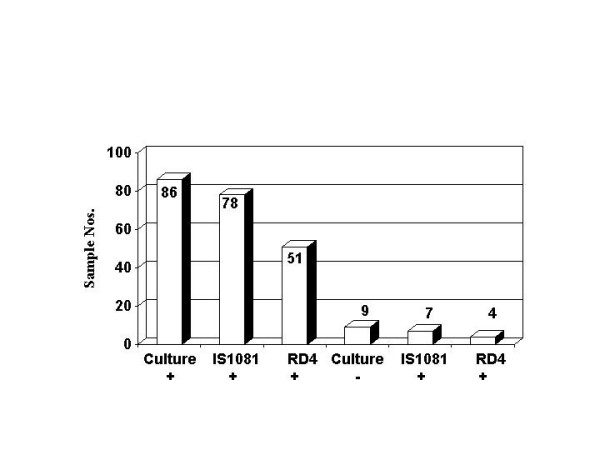
Summary of PCR and culture data from study 2. Numbers on the columns refer to the totals positive using each test. Compared to the "gold standard" of culture 78/86 (91%) were positive using IS*1081 *PCR and 51/86 (59.3%) were positive by RD4 PCR.

A formal test of this procedure was performed by assay of a further 95 lymph-node extracts from tissues with VL. Of these, 86 were subsequently shown to be culture positive for *M. bovis*, 9 were negative. Again, all extracts were PCR positive for bovine DNA (*cyt b*). Seventy-eight of the culture positives were IS*1081 *PCR positive (91%). Seven of the 9 culture negatives were also IS*1081 *PCR positive (Figure 2). Of 8 culture positive cases which were PCR negative for both IS*1081 *and RD4, 4 were reported as typical of bTB with acid-fast organisms, one was reported as atypical in appearance and 3 were not done due to insufficient tissue. The RD4 method detected 51 (59.3%) of culture positives and 4 of the culture negatives. In both studies all RD4 positives were also positive by IS*1081 *PCR.

### Study 2 Histology

Sixty-seven of the 86 samples (78%) which were *M. bovis *culture positive were reported as typical of bTB with presence of acid-fast mycobacteria. Ten of the remainder were described as atypical in appearance. No histology report was filed on the remaining 9 cases as there was insufficient tissue available. Morphological appearances in 3 of the 9 culture negative cases were reported as typical of bTB with acid-fast bacilli. Three were reported as atypical of bTB, one was reported as actinobacillus and no report was filed in 2 cases.

### Study 2 Comparison of PCR with histology

Of the culture positive cases reported as typical of bTB with acid-fast staining, 65/67 (97%) were IS*1081 *PCR positive and 40/67 (59.7%) were RD4 positive. Ten cases were atypical in appearance and acid-fast negative. Of these 10/10 (100%) were IS*1081 *positive and 9/10 (90%) were RD4 positive. Histology was not performed in 9 cases due to lack of tissue and of these, 6/9 (67%) and 5/9 (55.6%) respectively were PCR positive by IS*1081 *and RD4.

### Quantitation of VL samples using QPCR

In a small pilot study, real-time QPCR was used to measure *M. bovis *DNA in cattle extracts. Ten IS*1081 *PCR positive VL extracts were measured. The results are shown in Table 1. A wide variation in mycobacterial count was apparent. The use of IS*1081 *PCR for quantitation assumes that field strains of *M. bovis *contain the same copy number of IS*1081 *elements as reference strain used in preparation of the standards. This is a reasonable assumption for UK isolates and the greater majority of *M. bovis *strains likely to be encountered [23].

**Table 1:** Quantitation of *M. bovis *DNA in cattle VL samples

*Sample*	*VLA AF number*	*M. bovis genome equivalents*.	*RD4 PCR*
1	61/3188/03	2.54 × 10^6^	+
2	61/3461/03	10^4^	+
3	61/3462/03	910	+
4	61/3609/03	486	-
5	61/3689/03	3,500	+
6	61/3692/03	1,114	+
7	61/3694/03	910	+
8	61/3709/03	3.606 × 10^5^	+
9	61/3712/03	2,405	+
10	61/3714/03	2.393 × 10^4^	+

### Spoligotyping of VL extracts from studies 1 and 2

*Study 1*. All but 2 of the 98 culture-positive samples from the first study were successfully spoligotyped from cultures using the standard protocol. Four spoligotypes, 9, 17, 11 and 25 accounted for approximately 80% of the UK strains. The remaining isolates were spoligotypes 22 (6.1%), 10 and 20 (both 4.1%) and 15 (2%). Two spoligotypes, 35 and 81, occurred once each.

Of 47 VL DNA extracts spoligotyped directly from study 1, 24 (51%) generated recognisable patterns. These were all cases PCR positive by both RD4 and IS*1081*. A further 5 produced recognisable spoligotypes but with missing spacers, probably reflecting a form of allele "drop-out". The remaining 18 samples failed to give more than a few spacers and no type could be determined despite the fact that 13 of these were also PCR positive for IS*1081 *and RD4. Spoligotyping of 38 samples from study 2 was performed at VLA using the standard protocol (35 cycles of amplification). Of these just 11 (29%) yielded a recognisable pattern, the remainder producing only a partial fingerprint. Therefore, although a greater success rate was obtained with the modified PCR procedure, these findings were suggestive of poor DNA quality or quantity in both studies.

## Discussion

We have used a sensitive PCR based assay to screen cattle lymph nodes with VL suggestive of infection with *M. bovis *and have compared the findings with the gold standard method of diagnosis using routine culture and with histology. The IS*1081 *PCR was found to be extremely sensitive as judged by detection of partially-purified mycobacterial DNA, detecting less than one genome copy. This is almost certainly due to the multi-copy nature of the target [23]. The RD4 PCR was approximately 10 times less sensitive but still able to detect the equivalent of 5 organisms when using purified DNA.

The first study of VL extracts, in which only 70.4% of culture positives were also positive by IS*1081 *PCR, was therefore surprising. We judge that the measures taken to overcome PCR inhibition were effective. We base this on the successful amplification of the *cytb *housekeeper gene product in all cases, on the formation of primer-dimer in reaction tubes negative for IS*1081 *as well as on the IS*1081 *PCR success rate in study 2 (91% cf. culture). Hemi-nesting of the first-round IS*1081 *products did not greatly increase the pick-up rate by more that 5%, to 75.4%. This suggests that the first-round was already optimised and that DNA template may have been limiting. The second PCR was specific for *M. bovis *and used primers flanking the region of difference which defines this mycobacterium [24]. In study 1, the RD4 assay detected 50% of *M. bovis *culture positives. As the method amplifies a single-copy target, some reduced sensitivity is to be expected compared to IS*1081 *PCR. However, sensitivity of both methods was disappointing given the minimum detection limits determined for amplification of partially-purified genomic mycobacterial DNA.

It is well accepted that the initial processing of mycobacterial samples can be problematical compared to eukaryotic cells. We therefore embarked on a series of experiments to determine the cause in this instance. Key amongst these was the observation that the recovery of DNA from intact cultured *M. bovis *cells added to crude homogenates could be as low as 22% of the expected yield. Bead beating, sonication and freeze thawing in liquid nitrogen were examined for their ability to increase this yield. Using QPCR it was found that simple "snap" freezing 3 times in liquid nitrogen increased recovery of *M. bovis *DNA from intact cells by a factor of 3. Subsequently, the routine NucliSens™ extraction procedure was modified to include this step and the result compared with culture using a further 95 samples.

Results from the second study indicated a far greater overall sensitivity of the IS*1081 *screening PCR when the freeze/thaw cycles were performed. The IS*1081 *sensitivity rate for culture positives rose to 91% (78/86 cases). The detection rate with the RD4 PCR also increased, to 59.3% (51/86 cases). As found for study 1, both methods also detected positives amongst the culture negative cases (7/9 for IS*1081 *and 4/9 for RD4). This probably reflects amplification of mycocterial DNA from non-viable organisms, rather than contamination. All extracts prepared in study 2 were again positive for bovine DNA. The 91% sensitivity achieved with IS*1081 *linked with a modified extraction protocol, shows that this PCR would be a feasible addition to culture when rapid results are required. It is likely that increase in sensitivity reflects greater recovery of mycobacterial DNA. We speculate this stems from either improved lysis of the mycobacterial cell walls or greater dissociation of the DNA from particulate matter in the crude homogenates, allowing improved recovery in supernatants after centrifugation.

The sensitivity of PCR was found to be greater than histology (91% vs. 78%) and there was no evidence to suggest that most of the PCR negative samples were those with few or absent acid-fast bacilli. Indeed, of 10 samples reported as atypical of bTB, all were positive by IS*1081 *PCR. In light of these observations there may be a case for reviewing the role of histology in the diagnosis of bTB, possibly through comparison of the technique with quantitative PCR and culture of material with visible lesions.

Examination of the literature reveals the importance of the extraction step. Mycobacteria present some well recognised problems not generally encountered with other bacteria or eukaryotic cells and these are related to the robust mycobacterial envelope [25,26]. PCR methods should be assessed as a combination of both DNA recovery and the PCR. The extraction procedure should deliver effective lysis of mycobacteria, good recovery of the DNA from a complex mixture of tissue debris and lastly, removal of PCR inhibitors. A number of studies have addressed the problem of initial processing of mycobacterial samples and a number of procedures are described. These range from simple boiling and centrifugation [27], trapping of DNA on chelex resin [28], bead-beating [29], sonication [30], enzymic digestion [31], sequence capture [19,32], commercial kits with lysis reagents [33] and combinations of these various approaches [28]. Several of these studies have compared procedures, often with differing conclusions. The literature is particularly complex on this subject and reflects the fact that groups have compared different versions of the same general method, tested different samples and assessed recovery using different criteria. For example, the ability to obtain a PCR product or not on known culture positives [27], the absorbance (OD) of recovered DNA [29] or real-time PCR to quantitate the DNA [33]. It seems fair to conclude that there are a number of different methods which can be made to work effectively if sufficient steps are included to satisfy the three main criteria listed above [34]. For routine diagnostic use, there are the further considerations of ease of use, potential for automation and cost.

Silica-based methods of DNA extraction [35] have been widely evaluated and found to be one of the most efficient with columns generally more efficient than slurries [36]. However, homogenates from the cattle VL samples tested were lipaemic and noted to block resin-containing columns; therefore slurries of silica were used in the present study. The guanidinium buffer was shown to inactivate mycobacteria after overnight exposure, allowing processing of samples to take place outside a category 3 containment laboratory. Minor modifications were made to the standard wash steps (see Methods) to keep the silica free-flowing. Whilst the NucliSens™ kit is generally good at removal of PCR inhibitors, these were still encountered in VL extracts, and so these were routinely diluted to overcome their effect and additional *Taq *polymerase added.

QPCR using the IS*1081 *and RD4 methods was achieved by the simple inclusion of the DNA intercalating dye SYBR Green in the master mixes. SYBR Green provides an economical means of performing QPCR and additionally allows monitoring of conventional PCR. It was used routinely to optimise assay parameters, to follow product development and to ensure reliability of contamination measures. Melt analysis of amplicons formed at the end of the run was generally found to be a good indicator of outcome but we would still recommend gel electrophoresis to confirm correct product size as occasionally negative samples which generate a "ladder" of non-specific products are encountered and these can complicate interpretation of the melt profiles. Alternatively, Taqman™ versions of the assays could be easily devised and these would preclude the need for melt analysis or gel electrophoresis.

In a proof-of-principle study we applied QPCR to 10 VL extracts using the IS*1081 *method. The results (Table 1) showed mycobacterial copies in extracts ranging from a few hundred genome equivalents up to over 2 million. The paucibacillary nature of some of the extracts probably explains in part the low sensitivity of the RD4 method as only 5% of any extract was assayed in any PCR assay. Dilution to overcome PCR inhibition would have further reduced this to as little as five genome equivalents in some cases (e.g. sample 4, Table 1) bringing it near the limits of detection. Therefore, our results suggest QPCR is best undertaken with IS*1081 *PCR or other multi-copy targets.

Attempts at direct spoligotyping indicated that this technique could not be relied upon to produce a full fingerprint in up to 50% of VL samples. We consider that the failure to generate a full pattern in these cases might be due to either poor quality or quantity of DNA in some tissue extracts or possibly, a combination of both. Spoligotyping is a form of multiplex PCR in that multiple loci must amplify to obtain a complete fingerprint and multiplex PCRs tend to be less sensitive than those amplifying single loci [37]. The quantitative PCR study on VL specimens showed that the numbers of organisms in some extracts was limited to a few hundred and when dilutions are taken into consideration then DNA in aliquots would be near or at the limits of detection of the PCR technique.

The use of primers flanking deletion regions in the *M. bovis *genomes, which encompasses classic *M. bovis *subspecies such as *M. bovis caprae, M. pinnipedii *and antelope and other animal isolates, ensures a degree of specificity not generally attainable for detection of pathogen DNA particularly if sampling from problem sources such as faeces, soil or other environmental material. We have previously used methods which exploit these one-way deletion events for detection and categorising isolates within this lineage, as have others [6,38,39]. An assessment of both IS*1081 *and RD4 methods on environmental samples is planned as part of a new DEFRA initiative in late 2007. This will follow OIE criteria for validation and will be a stringent test of the IS*1081 *PCR.

## Conclusion

We suggest that the IS*1081 *PCR is a good candidate assay for routine screening of cattle lymph nodes and other tissues for *M. bovis *or other MTB complex infection. Efficient DNA extraction is crucial to the success rate of PCRs applied to such tissues, where mycobacterial numbers can be low. The resilient mycobacterial cell wall, the presence of tissue debris and the paucibacillary nature of some VL tissue may all contribute to this problem. Any of these factors may restrict application of other more discriminant typing methods.

Further quantification of numbers of bacilli in cattle lymph nodes from VL and NVL cases is required to determine the proportion of samples in which PCR is likely to be beneficial to diagnosis. The IS*1081 *PCR may also be useful for reducing the total number of samples cultured. Moreover, as the sensitivity of PCR was greater than histology (91% cf. 78%) and results can be available within a few days, there may be a case for replacing histology with a molecular method.

Confirmatory and genotyping tests, like the RD4 PCR (or multiple RD deletion typing) and other multiplex methods are unlikely to achieve a useful sensitivity in paucibacillary specimens unless some way can be found of improving assay sensitivity, such as single tube nested methods. Similarly, spoligotyping and more discriminant typing methods like VNTR are likely to be restricted to multibacillary tissues or to DNA purified from cultures unless some means can be found of improving method sensitivities for routine diagnostic use.

## Methods

### Samples

Two separate studies were undertaken on cattle lymph nodes with VL and the results compared. The samples were all from skin test reactors, none were from slaughterhouse cases.

Study 1 comprised of 109 lymph node tissue samples with VL assayed for *M. bovis *in 2003 by the VLA, Weybridge. Based on the PCR results obtained from study 1, minor modifications were made to the DNA extraction procedure and the effects of these were evaluated in a second study. Samples were stored before testing at -20°C.

Study 2 comprised of a further 95 VL samples received during 2004. These were stored at 4°C before extraction.

### DNA extraction method for bovine tissue samples

Tissue homogenates were prepared using NucliSens™ kits from bioMérieux. This system relies on initial cell lysis in a 5M guanidinium buffer containing detergents followed by trapping of released DNA onto silica beads and partial purification with the DNA isolation reagents. This process is based on the original Boom method [35].

Sterile scissors and forceps were used to sort through the tissue samples, discarding as much fat as possible. A piece of lesioned material was placed in a small pestle and mortar (90 mm diameter) and 9 ml NucliSens™ buffer was added together with 5 cm^3^of sterile glass beads. The tissue was ground to a suspension and then transferred back to the 9 ml buffer tube ready for DNA extraction.

Study 1. The manufacturers' instructions were followed with modifications to cope with cattle tissue samples. The 9 ml size lysis buffer tube was used. Homogenates were heated in a boiling water bath for 5 mins. and then stored at -20°C until extraction was continued. When thawed, samples were centrifuged at 2000 rpm (1,100 × g) for 5 mins. and the supernatants transferred into 15-ml conical centrifuge tubes (Corning). Silica suspension (50 μl) was added and the tubes were mixed on a rotating wheel (Stuart) for 30 mins. After further centrifugation at 2000 rpm (1,100 × g) for 5 mins., the supernatants were discarded into disposable 50 ml Falcon tubes ready for safe disposal of the guanidine thiocyanate (GUSCN) lysis buffer.

The silica pellets were washed twice with each solution in turn according to the kit instructions (GUSCN wash buffer, 70% ethanol and acetone) except that double volumes of each wash solution were used (2 ml). This was necessary to remove tissue debris and lipid residues from the silica, allowing it to form a suspension upon vortexing.

The acetone was decanted and the silica allowed to dry at room temperature (RT). DNA was eluted from the silica with 100 μl of the supplied elution buffer. This step was performed at 60°C for 10 mins. to aid DNA recovery. The tubes were vortexed, centrifuged and supernatants transferred to 0.5 ml low-retention flat-cap tubes (Alpha Labs). Dilutions of the extracts (1/5, 1/10 and 1/20) were prepared to overcome PCR inhibition and 5 μl of each assayed.

Study 2. This was similar to study 1 except that after heat-killing for 5 mins. in the boiling bath, extracts were "snap" frozen 3 times in liquid nitrogen to assist with disruption of mycobacteria. DNA was then extracted as described for study 1.

### DNA extraction method for mycobacterial reference strains

Colonies of *M. tuberculosis *H37Rv, H37Ra, CDC1551 and *M. microti *(NCTC NC08712-04, NC8710-04 and NC8337-01) were removed from the growth media, heat-killed for 5 mins. at 100°C and genomic DNA partially purified using the bioMérieux NucliSens™ kit following the manufacturers' instructions. Isolates of MOTT supplied by VLA Weybridge were heat-killed and cells and other debris pelleted by centrifugation at 10,000 × rpm (5,500 × g) for 10 mins. and the supernatant used directly in PCR experiments.

### Measures to prevent contamination

Throughout the extraction procedure great care was taken to avoid cross-contamination between samples. Gloves were changed frequently. The overall strategy emphasizes the use of physical barriers with separate areas for extraction, PCR set-up and product analysis. At Imperial College this comprises a two-laboratory three-workstation method. Sample extraction and PCR set-up take place at separate areas of laboratory one and PCR product analysis in laboratory two. Filtered air is ducted and diffused separately into both laboratories with extraction through the second laboratory, minimizing the chances for air-borne contamination of laboratory one. Separate sets of pipettes were used for PCR set-up and product analysis. The former was stripped and cleaned in detergent and ethanol before each experiment. Filter tips were used routinely. Surfaces and equipment in contact with sample tubes (centrifuges, rotors, mixers, etc.) were cleaned before each assay.

### Tissue culture

Tissue was also taken for routine culture. Tissue slices (3 mm) were placed in a large mortar (110 mm diameter) and ground with 5 cm^3 ^sterile glass beads. Thirty ml of 5% oxalic acid was added to the mortar, the contents mixed, left for 10 mins. and then centrifuged for 10 minutes at 1100 × g. The acid supernatant was discarded; the tissue homogenates re-suspended in 20 ml of 0.85% saline and the centrifugation step was repeated. The saline supernatant was discarded and the residues re-suspended in a further 10 ml of 0.85% saline. Aliquots of this final suspension (300 μl) were inoculated onto six slopes of media comprised of 1 LJ base, 1 LJG, 1 LJP and 3 modified 7H11 [40]. The media were incubated for 6 weeks at 37°C.

### Histology

Approximately 1 cm^3 ^portions of lesioned lymph node material (study 2) were placed into sterile universals containing buffered formalin for routine histological examination. Histology data was not available on samples included in study 1, which were part of a large backlog of specimens accumulated after the foot and mouth outbreak of 2001.

### Heat inactivation trial of *M. bovis *with NucliSens™ lysis buffer

This experiment was carried out to determine the efficacy of the lysis buffer with or without heat treatment in killing M. *bovis *isolated from cattle VL tissues.

A confirmed VL tissue sample (AF61/2834/02) was homogenised using a pestle and mortar and 18 ml of NucliSens™ lysis buffer was added at RT. The suspension was then pipetted back equally into two 9 ml tubes to provide a heated and unheated control sample. Additionally, a 1 ml aliquot of viable *M. bovis *AF61/2122/97 (6 × 10^7 ^CFU/ml) was added to two tubes of 9 ml NucliSens™ lysis buffer again for heating and an unheated control. The heated samples were then placed in a water bath at 95°C for 5 minutes. 300 μl of each treated sample was then inoculated onto 4 labelled 7H11 slopes. Four aliquots (300 μl) of AF61/2122/97 (untreated) were also inoculated onto 7H11 slopes as a positive control. The slopes were incubated at 37°C for 6 weeks.

### PCR

Three PCR tests were routinely applied to all presumptive cases of *M. bovis*. The primer sequences and cycling parameters are shown in Table 2. The first of these was a housekeeper gene for cattle DNA. The chosen target was the bovine mitochondrial cytochrome b gene (*cyt b*) and specific primers were selected to amplify a 359 bp product from this region (accession No. D34635). This product contains a 307 bp variable region and restriction fragment length polymorphism (RFLP) analysis permits identification of species if required [41]. The *cytb *method was run to ensure recovery of bovine DNA and removal of PCR inhibitors.

**Table 2:** PCR primers and parameters

*PCR*	*Primer*	*Sequence*	*[Mg] mM*	*Anneal temp*.	*Amplicon size (bp)*	*T*_*melt *_*Celsius*
Bos taurus	cytb F	5'-CCATCGAACATTTCATCATGATGGAA-3'	2.0	64	359	95
	cytb R	5'-GCTCCTCAGAATGATATTTGTCCTCA-3'				
IS1081	F2	5'-CTGCTCTCGACGTTCATCGCCG-3'			135	94
	R2	5'-GGCACGGGTGTCGAAATCACG-3'	2.0	58	113	
	R3	5-TGGCGGTAGCCGTTGCGC-3				
RD4 flanking	F1	5'-AATGGTTTGGTCATGACGCCTTC-3'		58	176	92
	F2	5-TGTGAATTCATACAAGCCGTAGTC-3	2.0		142	
	R1	5'-CCCGTAGCGTTACTGAGAAATTGC-3'				

The second test was used to screen for MTB complex mycobacteria and used primers to the core region of the multi-copy element IS*1081*, generally present in 6 copies [23]. Thirdly, to confirm presence of classical *M. bovis *in cattle VL extracts we used primers which flanked a region of difference (RD) in the bovine lineage known as RD4 [42]. The use of flanking primers ensured that PCR products were formed only if the deletion was present.

**Figure 1:**
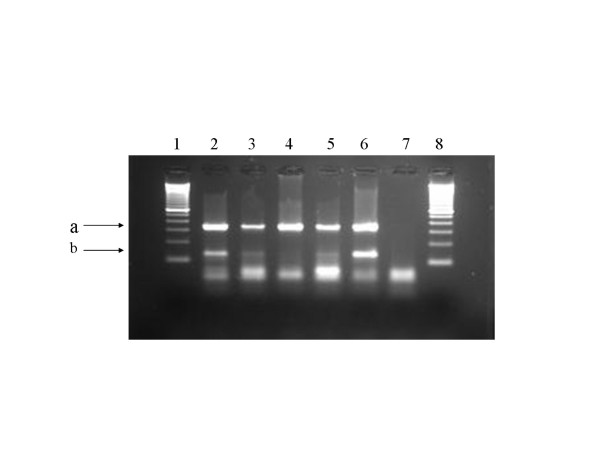
Gel electrophoresis of multiplex PCR products (IS*1081 *and *cytb*) run on 3% agarose gel. Lanes 1 and 8: 100 bp DNA size markers. Lanes 2–6: samples of cattle LN with VL. All show amplification of bovine mitochondrial DNA (*cytb *product, arrow a) and those in lanes 2, 3, 5 and 6 are positive for the IS*1081 *product of 135 bp (arrow b).

Size differences between PCR amplicons and identical magnesium requirement allow the methods to be combined as a multiplex test if required (e.g. Figure 1). In the present study they were applied individually to maximise sensitivity. Template blanks (water) and extraction controls, consisting of lysis buffer minus tissue extract, were included in every assay.

"Hot-start" PCR was performed in a final volume of 25 μl using the Corbett Research RotorGene 3000 real-time platform and the Excite Core kit (BioGene). This is a convenient uracil-N-glcosylase-ready kit which permits restriction of any carryover amplicons with uracil glycosylase, should this be required. All primers were used at a concentration of 25 pmoles/tube. SYBR Green dye (BioGene) was included in the PCR master mix at a final dilution of 1/55,000 of the stock. This dilution was found optimal in preliminary experiments and allowed the reactions to be followed on the Corbett platform. The recommended *Taq *polymerase was increased from 0.5 U to 1.5 U per reaction to overcome PCR inhibition. Annealing temperatures were first determined using a gradient block on a PCR Express thermal cycler (Hybaid Ltd). Magnesium optima were found using the RotorGene 3000 real-time PCR platform (Corbett Research). After an initial denaturation step (8 min at 95°C), 45 cycles of amplification were performed as follows: denaturation at 95°C for 10 s, annealing at optimal temperature (range 54 – 64°C) for 30 s, extension at 72°C for 20 s and acquisition of fluorescent signal at 85°C. At this temperature, most primer-dimers were not seen during the run. A final extension was performed at 72°C for 2 min. Melt analysis was performed at the end of the run to screen for positive samples using the RotorGene software. Familiarity with the melting characteristics of the 3 PCR products enabled positives to be readily identified at this stage but for confirmation an aliquot of each product was run out on 3% agarose for comparison with a 100 bp DNA ladder (Invitrogen).

### Quantitative real-time PCR (QPCR)

A quantitative version of the IS*1081 *PCR was developed for comparing copies of MTB complex genomes recovered from different VL samples. When this quantitative PCR assay was run on the Corbett Rotor-Gene 3000, VL extracts were assayed in duplicate and DNA standards were run in triplicate. The standard consisted of 5 μg of *M. bovis *DNA, strain AF 2122/97 which was partially purified using the bioMérieux kit from culture material held at the VLA reference laboratory. Serial dilutions of this stock (over the range 10^-1 ^to 10^-6^, 2,500 pg/5 μl to 0.25 pg/5 μl) were prepared in 1x TE buffer containing transfer tRNA (tRNA, Sigma) as a carrier at a final concentration of 245 μg/ml. Concentration of DNA in the stock was determined using a Varian Cary 50 UV-VIS spectrophotometer (Varian Inc. Walnut Creek, California, USA). To maximize consistency between runs, each standard (200 μl) was sub-aliquoted into 20 μl volumes and stored at -20°C. One set of standards was thawed before use and any remnants discarded. To further minimise loss of DNA onto plasticware, low retention plastic micro tubes (AlphaLabs) were used throughout for storage of extracts and for conventional and quantitative PCR. Quantitation analysis was made by interpolating cycle threshold (ct) values of samples against those for the standards using the Rotor-Gene software.

#### Spoligotyping

PCR-based typing (Spacer-OLIGOnucleotide typing – 'spoligotyping') was performed at VLA Weybridge on all positive cultures identified at week 6. The standard protocol of Kamerbeek was followed [16]. Spoligotyping was also performed on 47 DNA extracts prepared for study 1 at Imperial College using a modified procedure in which the cycle number was increased from 35 to 43 and additional *Taq *polymerase was added (1.5 U cf. 0.5 U). A further 38 extracts prepared for study 2 were spoligotyped at VLA using the standard protocol. Authoritative names for spoligotype patterns were obtained from the *M. bovis *spoligotype database world wide web site [43].

### Validation of the IS*1081 *and RD4 PCR methods

Specificity testing was undertaken on a variety of reference and field isolates of the *M. tuberculosis *complex held at Imperial College London and VLA Weybridge. The strains tested included *M. canetti *(Somalia), *M. tuberculosis *H37Rv and H37Ra and CDC 1551. The methods were tested against three reference strains of *M. microti *(NCTC NC08712-04, NC8710-04 and NC8337-01) and on 30 field isolates of *M. microti *from diverse animal species (spoligotypes 18, 28, 32 & 34). A number of strains causing disease in animals were also used for PCR validation. These included the sequenced reference strain *M. bovis *AF2122/97, *M. bovis *BCG Pasteur and a panel of 10 *M. bovis *isolates with different spoligotypes commonly isolated in the UK (Table 3). Additionally, the RD4 PCR was tested on an isolate from a fur seal, now recognized as a distinct member of the complex, *M. pinnipedii *[44].

**Table 3:** VLA spoligotype panel used in validation of PCR methods

*VLA sample*	*VLA strain AF No*.	*Spoligotype*	*International name*
1	61/3558/00	9	SB0140
2	61/1121/01	17	SB0263
3	61/2145/00	12	SB0271
4	61/5415/00	11	SB0274
5	21/0140/01	20	SB0145
6	61/3979/00	13	SB0273
7	61/0288/01	22	SB0673
8	61/5488/00	10	SB0272
9	61/0681/01	25	SB0124
10	61/1307/01	35	SB0134

Mycobacterial species other than tuberculosis (MOTT) were also used in the assessment of PCR specificity. The strains tested included *M. paratuberculosis *(NCTC 8578), *M. xenopi *(NCTC 10042), (*M. gordonae *(NCTC 10267), *M. fortuitum *(NCTC 10394), *M. intracellulare *(NCTC 13950), *M. marinum *(NCTC2275) and *M. avium *(NCTC 8559).

### Minimum detection limits

The minimum detection limits for the IS*1081 *and RD4 methods were compared by testing serial dilutions of genomic DNA partially-purified from M. *bovis *strain AF2122/97 diluted in TE buffer, pH 7.0. Initial concentration of the DNA working stock was determined by spectrophotometry as described above for QPCR.

## Authors' contributions

PCR methods for *M. bovis *and bovine mitochondrial DNA were developed by GMT. Initial sample processing, the heat inactivation trial and supervision of tissue culture of cattle lymph nodes was performed at VLA Weybridge by DRW. Spoligotyping at VLA and Imperial College was undertaken by SP and GMT respectively. KJ, RGH and GMT contributed equally to design of the studies and in the preparation of the manuscript. All authors have read and approved the final manuscript.
